# Items

**Published:** 1882-10

**Authors:** 


### Items.

CERTIFICATES of Practice, entitling the holders to practice medicine and surgery or midwifery in Illinois, have been issued to the following named practitioners by the State Board of Health :

E. L. M. Bristol, M.D., Chicago, Ill.; diploma of Jefferson Medical College, Philadelphia, Pa.

Albert Beasley, M.D., Grand Tower, Ill.; diploma of Missouri Medical College, St. Louis, Mo.

Julius H. Dietrick, M.D., Chicago; diploma of Swiss Medical Council, Zurich, Switzerland.

Albert B. DeLiplay, M.D., Chicago; diploma of Medical Department, University of Michigan, Ann Arbor, Mich.

Wm. A. Johnston, M.D., Peoria; diploma of Medical Department University of Pennsylvania, Philadelphia, Pa.

Charles H. McGorray, M.D., Chicago; diploma of Medical Department University of Michigan, Ann Arbor, Mich.

A. J. McIntyre, M.D., Thompsonville, Ill.; diploma of Medical Department University of Michigan, Ann Arbor, Mich.

Patrick McCambridge, M.D., Springfield; diploma of Missouri Medical College, St. Louis, Mo.

J. M. Patton, M.D., Oak Park, Ill.; diploma of Medical Department University of the City of New York.

Isaac N. Ruddell, M.D., Charleston, Ill.; diploma of Medical Department University of Louisville, Ky.

Thomas C. Rivers, M.D., Chicago; diploma of Medical Department University of Louisiana, New Orleans, La.

Ernst P. Raab, M.D., Highland, Ill; diploma of Medical Department University of Pennsylvania, Philadelphia, Pa.

M. M. Robinson, M.D., Enfield, Ill.; diploma of Kentucky School of Medicine, Louisville, Ky.

M. F. Spaulding, M.D., Kimmswick, Mo.; diploma of St. Louis Medical College, St. Louis, Mo.

Carl F. Sandberg, M.D., Chicago; diploma of Christiana University, Norway.

Dr. H. Saguin, Amboy, Ill.; certificate of twenty-four years' practice.

Dr. John A. Simmerman, Lane, Ill.; certificate of twenty-seven years' practice.

Edward K. Tyler, M.D., Illinois City, Ill.; diploma of the Medical Department, Iowa State University.

Frank A. Wyanet, M.D., Pullman, Ill.; diploma of the Medical Department University of Michigan, Ann Arbor, Mich.

David A. White, M.D., Oak Grove, Ill.; diploma of the Ohio Medical College, Cincinnati, O.

Charles F. White, M.D., Wayne City, Ill.; diploma of the Missouri Medical College, St. Louis, Mo.

Minnie E. Buchner, midwife, Chicago, Ill.; certificate of the Missouri School of Midwifery.

Elizabeth Brumer, midwife, Piopolis, Ill.; sixteen years' practice.

Elizabeth Koester, midwife, Plum Hill, Ill.; sixteen years' practice.

Johanna Mueller, midwife, Chicago; certificate of examination.

Karoline Novetney, midwife, Chicago; certificate of the University of Prague.

Elizabeth Richardson, midwife, Bluff City, Ill.; sixteen years' practice.

---

STATISTICS OF INSANITY IN THE UNITED STATES.—Dr. C. L. Dana gives (*Journal of Nervous and Mental Disease*, April, 1882) the following statistics regarding the insane in this country: We had in 1880, in round numbers, 89,000 to 96,000 insane, which gives a ratio of 1-570 (1-520) of the population. The census ratio in 1860 was 1-1,310; in 1870, 1-1,100; in 1875, 1-953. Our population increased in the decade 1870 to 1880 about 26 per cent., while our insane population has apparently increased over 100 per cent. As regards the distribution of insanity and of its increase, the proportion of insane is greatest in New England, where the ratio is one to 357. Here, however, the rate of increase is becoming slower. In proportionate number of insane, after the New England and Pacific States, come the Middle States (1 to 446); then the Western (1 to 520), and then the Southern (1 to 780). As regards asylum accommodations, in 1881 we had 74 State and 14 large private asylums, with a capacity, approximately, for 31,900, but holding 39,145. At a very low estimate, therefore, our asylums are overcrowded to the extent of 10,000 patients, while there are about 50,000 who are not in any asylum at all. Regarding the cost of asylums, we have about $40,000,000 invested in these institutions, at an average cost of over half a million apiece. It takes about $8,000,000 a year to run them, or $82,000 for each institution, not including interest. If we should add interest, the total annual expenditure for the care of the insane amounts to $12,000,000. The annual cost for each patient has been variously estimated at from $166 to $316.

THE MANAGEABLE ZONE OF ANÆSTHETICS.—Paul Bert has announced some discoveries of great value respecting the mode of action of mixtures of anæsthetic vapors and atmospheric air upon the animal organism. He applies the term manageable zone to the different degrees of admixture, rising from the proportion of anæsthetic which is insufficient to put to sleep, to the proportion which will cause immediate death. In the case of chloroform and ether, the mortal dose appears to be exactly double the minimum anæsthetic dose. Anæsthesia takes place in the middle of the manageable zone very rapidly, and without danger, so that the animal may be left for two hours in the anæsthetic atmosphere without any one being concerned about him. The manageable zone of ether is a little more than three times as wide as that of chloroform, while that of the protoxide of nitrogen is considerably wider that of ether. Here we see why ether is less, and the protoxide of iron still less, dangerous than chloroform.

DR. BLISS has appealed directly to the board of audit to be allowed $25,000 for his services as Mr. Garfield's physician. He claims his practice was worth $1,500 a month, and this was lost by reason of his attentions to the President. If he was not more successful with his other patients than he was with the President, those who recovered because he had to leave them might feel like "chipping in" to help the government get rid of him.—*Daily News.*

EPIDEMIC CHOLERA.—300 deaths from cholera occurred in one day at Manila, Phillipine Islands, lately. 294 of the victims were natives. A cable dispatch from Japan, August 29, stated that out of 755 cases of cholera within twenty days at Yokohama, 572 cases proved fatal. At Tokio about 80 cases and 50 deaths have occurred daily.

PROF. J. ADAMS ALLEN, President of Rush Medical College, has returned much improved in health from his prolonged tour through Europe, and has taken his residence at the Woodruff House.

Prof. Allen has removed his office to 125 State St.

ELECTRICITY is becoming a frequent cause of death. Two men, while attempting to climb the railing of the Tuileries gardens at Paris, during a display of fire-works, August 8, caught hold of the electric wires used in illuminating the grounds, and both were struck dead instantly.

AT a meeting of the Philadelphia Academy of Natural Sciences, February 7, Dr. Leidy exhibited specimens of worms from the black bass. They were bright red, three to six inches long, and lived coiled up in the stomach and other tissues of the fish.

OPIUM DENS.—August 24, a raid was made by the Chicago police on six opium dens. Nine whites and six Chinamen were arrested. No females were found, and the white men were of a very ordinary class.

THE Siberian plague is appearing in the most widely separated quarters of European Russia. An epidemic of measles spread in Iceland in August.

